# One-Size-Fits All: A Scalable Solution to Formal Telemedicine Provider Training to Support the COVID-19 Pandemic Response

**DOI:** 10.3389/fped.2021.647458

**Published:** 2021-03-30

**Authors:** Dana A. Schinasi, Yuemi An-Grogan, Rebecca Stephen, Aric Shimek, Marisa Furney, M. Katie Bohling

**Affiliations:** ^1^Department of Pediatrics, Northwestern University Feinberg School of Medicine, Chicago, IL, United States; ^2^Telehealth Programs, Ann & Robert H. Lurie Children's Hospital of Chicago, Chicago, IL, United States; ^3^Department of Emergency Medicine, McGovern Medical School at UTHealth, The University of Texas Health Science Center at Houston, Houston, TX, United States

**Keywords:** telemedicine, telehealth, COVID-19, training, education

## Abstract

**Introduction:** Formalized training in telemedicine addresses barriers to provider adoption and engagement and assures a level of competence for independent practice. We previously developed a blended-model training program, customizable according to role and specialty; this method of training was not feasible in the pandemic response. We describe the development and implementation of a multi- and interdisciplinary telemedicine provider training program enabling the rapid scaling of telemedicine at our institution.

**Methods:** An existing curriculum was pared down to a 1-h session delivered synchronously, covering the foundational components of telemedicine practice. Supplemental materials were available for asynchronous learning via the hospital intranet. Completion of training was required of all clinicians who practice telemedicine.

**Results:** We conducted 35 sessions for 1,070 providers over 12 weeks. Attendees included clinicians across numerous roles and specialties. Additional resources were created and available through the Telemedicine Virtual Handbook and housed in specific toolkits.

**Discussion:** Telemedicine training is necessary for consistent, competent practice of telemedicine in pediatrics. We describe a training process that can be easily replicated and rapidly deployed to providers of telemedicine across roles and disciplines. Combining a mandatory and brief synchronous provider training session with a repository of online resources creates a foundation for consistent practice, while allowing for more individualized resources accessible on demand. Standardized telemedicine training followed by mechanisms for ongoing professional practice evaluation allow institutions to ensure consistent and competent practice of telemedicine. Further study is needed to determine the best modality for training, and optimal assessment tools according to professional role.

## Introduction

Formalized training of providers in telemedicine is endorsed by the American Telemedicine Association (1), though is not yet standardized nor consistent across institutions. A formalized training program has the potential to address key issues previously cited as barriers to provider adoption and engagement in telemedicine, including ease of use and perceived usefulness of this modality of care delivery (2–5). Additionally, providers familiar with technology are more inclined to use telemedicine (6), a barrier which is overcome with exposure and hands-on training (7, 8). Health care providers tend to be reluctant to accept change (9), and the formal opportunity to have questions answered and concerns addressed by program leaders in real time through synchronous training may be a valuable means to overcome this. Additionally, training allows consistency in adherence to federal, state, and local standards, and ensures that providers are compliant in their practice. Finally, formal telemedicine training can be a pre-requisite to assignment of hospital telemedicine privileges; it has been our experience that formal telemedicine privileges help to expedite credentialing of our telemedicine providers externally with partner hospitals. Still, there are several deterrents to formal training in telemedicine: training requires specific resources in the form of time, personnel, and financial commitment, which may deter organizations from developing required, formalized programs.

There are currently over 30 training and certificate programs in telemedicine in the United States (10–12), with varying content and without standardization. Presently, academic programs lack guidelines for training, certification, and accreditation, although the American Association of Medical Colleges published its Telehealth Competencies (13) in September 2020, signaling an important step in the right direction. Development of a core set of competencies and standards is also underway at various health care organizations and national collaboratives. Still, with the recognition that *web*side delivery of care differs from care delivered in-person at the *bed*side, there are foundational components that each telemedicine provider should have a basic knowledge of and demonstrate competence in. These foundational components were determined based on our institutional experience and informed by existing literature (1), and include: local context and programmatic goals; legal and risk considerations; workflows; clinical considerations, including physical examination techniques and charting requirements; virtual presence, including webside manner overview; and technology overview, including hardware, software, and troubleshooting.

Adults learn best by experiential learning modalities, including small groups, hands-on practice, role-play, and simulation. These blended learning approaches have been increasingly employed in medical education, using a combination of face-to-face and online learning, augmented with other experiential modalities for education (14, 15). At Ann & Robert H. Lurie Children's Hospital of Chicago, medical educators and telemedicine program leaders collaborated to develop a comprehensive blended model training program, incorporating small group work, online content, and simulation-based training. This curriculum is customizable according to provider role and specialty, and its successful completion is a requirement for Telemedicine Privileges through our Medical Staff Office (MSO).

This method of training in telemedicine was not feasible during the pandemic response. COVID-19 necessitated widespread rapid deployment of a telemedicine curriculum to enable competent and independent practice of telemedicine, a modality of care that had not been widely practiced by pediatric providers at our institution previously. As we endeavored to rapidly train hundreds of providers during the pandemic response, we needed to adapt our existing training requirements to meet their basic educational and operational needs. Here, we describe the development and implementation of an easily reproducible and scalable telemedicine provider training curriculum reaching across the numerous specialties, roles, and practice settings at our children's hospital.

### Curriculum Development and Deployment

The existing customizable Telemedicine Provider Training curriculum was developed following Kern's six-step model for curriculum development: (1) problem identification and general needs assessment, (2) targeted needs assessment, (3) goals and objectives, (4) educational strategies, (5) implementation, and (6) concepts for evaluating the effectiveness of the curriculum (16). The educational effectiveness of this curriculum was assessed using checklists for observed simulations and anonymous web-based surveys for self-reported knowledge and attitudes assessment, finding improvement in knowledge and attitudes following training, specifically regarding workflow and processes, provider roles, and medicolegal issues; these results are yet unpublished.

Given the large scale and abbreviated timeframe to deploy telemedicine in response to the COVID-19 pandemic, we needed to adapt our existing training requirements to meet their basic educational and operational needs. Our approach in paring down the targeted training to a 1-h session covering key foundational components delivered synchronously to all clinicians regardless of role, discipline, or setting started with areview of each of the previous curricula utilized for existing programs, peeling away their specific workflows and clinical considerations. Through this exercise, we honed in on the foundational components relevant to all disciplines, roles, and practice settings, and used these to scaffold the 1-h Telemedicine Provider Training. We then layered in updated regulations, technology standards, and workflows related to our institutional ramp-up in response to the pandemic. Individual hands-on practice and additional role-specific workflow and operational discussions were at the discretion of individual service lines, based on their telemedicine utilization and plans. We then developed an institutional “Telemedicine Virtual Handbook” and published it on the Lurie Children's intranet as a dynamic, asynchronous resource with further detail on topics introduced in the structured training. In-person training was offered in the hospital conference center, where in-person attendance was capped to ensure compliance with safe social distancing recommendations. A virtual attendance option was available for those who were unable to safely attend in-person.

### Synchronous Training

We conducted 35 sessions and trained 1,070 providers over 12 weeks; 27 sessions were conducted with 791 providers trained in the first 4 weeks following the declaration of the national emergency. Those in attendance included a mix of physicians, advanced practice providers, psychologists, social workers, clinical nutritionists, genetic counselors, case managers, and more. They spanned the departments of Pediatrics (general and subspecialty), Surgery (general and subspecialty), Psychiatry, Rehabilitation Services (physical, occupational, and speech therapy), and Radiology. As resident and fellow trainees re-joined the clinical workforce, they, too, received formal telemedicine training. In total, 595 providers received Disaster Telemedicine Privileges through the MSO, which were immediately released, and lasted 120 days.

Specific content included within each of the foundational components of the synchronous telemedicine training are reviewed here. First, definitions and *local context* are provided, along with the sharing of institutional telehealth vision and programmatic goals. Federal, state, and local *legal considerations* are reviewed next to ensure that telemedicine providers are up to date on current policies and standards on provision of care and reimbursement for telemedicine services. *Risk management* is a previously cited concern to provider adoption of telemedicine; it is an essential component of training as it relates to scope of practice, malpractice coverage, limitations of physical assessment, and a review of resources or policies for action in the event of witnessing a situation concerning for child maltreatment. A review of *clinical considerations* includes an introduction to the observational physical examination, an overview of workflows, and an introduction of charting requirements. The *virtual presence* component reviews best practices for webside manner, introduces privacy and confidentiality as it relates to telemedicine, and conveys standards for professional appearance during an encounter. Regardless of specific technology (hardware and software) investment, the basic principles of *telemedicine technology training* are the same. These include an overview of equipment sanctioned for use; software platform(s) for video visit, image-sharing, and communication; discussion of network connections; and introduction of troubleshooting tips and resources.

### Asynchronous Training

Additional resources were created on the hospital SharePoint site, a customizable cloud-based content collaboration and management platform that houses the Lurie Children's intranet (17). These resources are located in the Telemedicine Virtual Handbook page of the intranet and are housed in specific toolkits. The *Provider Toolkit* includes electronic health record guides and tip sheets, a physical examination tip sheet, a library of videos developed by local colleagues on various telemedicine physical examination components, a visit checklist, guides for accessing interpreting services, as well as sample materials (e.g., patient/family pre-visit letter). The *Technical Toolkit* includes guides and tip sheets for troubleshooting hardware and software. The *Scheduling Toolkit* includes training videos for appointment schedulers as well as guides for helping patients and families prepare for their visit, including obtaining and troubleshooting internet access. The *Webside Manner Toolkit* houses best practices for on-camera presentation and overall virtual experience. Additional resources housed on the Telemedicine Virtual Handbook include mechanisms for support, important announcements, opportunities for information sharing, as well as a repository for resources for program evaluation and quality improvement. The Telemedicine Virtual Handbook remains a dynamic resource, easily accessible through the hospital intranet, with updates and additional content added as needed.

### Ongoing Quality Assessment

Telemedicine is an additional modality to support and augment clinical practice; as such, measures to assess the quality of care delivered should align with those quality measures for in-person practice. This can be readily accomplished in concert with an assignment of distinct Telemedicine Privileges through the MSO. In accordance with Joint Commission requirements, once privileges are attained, telemedicine providers enter the Focused Professional Practice Evaluation (FPPE) process, to assure the privilege-specific competence of the individual practitioner (18); at our institution, this entails a discrete number of encounters reviewed by the division head or medical director. Once the FPPE requirements are satisfied for telemedicine, the Ongoing Professional Practice Evaluation (OPPE) is overseen by the individual department or division clinical leadership, according to their usual practice. Additional measures for evaluation of quality were suggested according to established frameworks for telemedicine evaluation and measurement (19, 20), and are incorporated at the discretion of clinical leadership. Domains include activity data, medical/clinical knowledge, interpersonal/communication skills, professionalism, and systems-based practice. Ongoing evaluation is paramount to ensure that telemedicine programs reach patients equitably, are high-quality, and cost-effective.

## Discussion

Telemedicine training is necessary for consistent, competent practice of telemedicine in pediatrics. We describe a training process that can be easily replicated and rapidly deployed to telemedicine providers across roles and specialties. Combining a mandatory and brief synchronous provider training session with a growing repository of online resources creates a foundation of consistent practice, while also allowing for more detailed and individualized resources that can be accessed on-demand. In our experience, standardized telemedicine training followed by mechanisms for ongoing evaluation has allowed our institution to ensure consistent and competent practice of telemedicine; collection of data geared toward evaluation of the curriculum is warranted and is an area of future research. While further study is needed to determine the best modality for training and the optimal assessment tools according to professional role, a workforce trained in telemedicine is best poised to advocate for meaningful and lasting changes to improve access to care for our patients and families.

## Data Availability Statement

The original contributions presented in the study are included in the article/supplementary material, further inquiries can be directed to the corresponding author/s.

## Author Contributions

DS and YA-G developed the curriculum, drafted the initial manuscript, reviewed, and revised the manuscript. RS, AS, and MF reviewed and revised the manuscript. MB developed the curriculum and reviewed and revised the manuscript. All authors approved the final manuscript as submitted and agree to be accountable for all aspects of the work.

## Conflict of Interest

The authors declare that the research was conducted in the absence of any commercial or financial relationships that could be construed as a potential conflict of interest.
